# Prognostic factors in the decision-making process for sigmoid volvulus: results of a single-centre retrospective cohort study

**DOI:** 10.1186/s12893-022-01549-4

**Published:** 2022-03-14

**Authors:** Zoe Slack, Mohamed Shams, Raheel Ahmad, Roshneen Ali, Diandra Antunes, Abhishek Dey, Mahul Patel, Amanda Shabana, Giles Bond-Smith, Giovanni D. Tebala

**Affiliations:** Surgical Emergency Unit, Oxford University Hospitals NHS Foundation Trust, John Radcliffe Hospital, Headley Way, Headington, Oxford, OX3 9DU UK

**Keywords:** Sigmoid volvulus, Frailty, Emergency colon resection

## Abstract

**Background:**

Sigmoid volvulus is a common cause of emergency surgical admission. Those patients are often treated conservatively with a high rate of recurrence. We wondered if a more aggressive management might be indicated.

**Methods:**

We have reviewed data of patients diagnosed with acute sigmoid volvulus over a 2-year period. The primary endpoint was patient survival.

**Results:**

We analysed 332 admissions of 78 patients. 39.7% underwent resection. Survival was 54.9 ± 8.8 months from the first hospitalization, irrespective of the treatment. Long-term survival was positively influenced by being female, having a low “social score”, a younger age and surgery. Multivariate analysis showed that only being female and surgery were independently associated with better survival.

**Conclusion:**

Early surgery may be the best approach in patients with recurrent sigmoid volvulus, as it ensures longer survival with a better quality of life, regardless of the patient’s social and functional condition.

## Background

Sigmoid volvulus has been formally reported by Von Rokitanski in his book “A Manual of Pathological Anatomy” in 1848, but many centuries before, the Papyrus Ebers already described “twist in a bowel that if left untreated will rot and the abdomen will become stiff, distended and painful, unless treatment is given to evacuate the bowel immediately” [1].

In the western world sigmoid volvulus affects older males who are institutionalized and may have neurological deficit making them less preferable surgical candidates [2, 3]. Other pre-disposing factors include chronic constipation, neurological disease, anatomical predisposition and adhesions [4, 5]. Complications include ischaemia, gangrene and perforation which can result in a surgical emergency. Definitive treatment is surgical, but first line treatment is via endoscopic devolution with or without placement of a rectal tube. After non-operative management recurrence occurs in 50–90% of cases [3, 4] carrying a mortality of 7–20% [5–7]. Due to the high risk of recurrence, the American Society of Colon and Rectal Surgeons (ASCRS) publicized guidelines in 2016 stating that patients should undergo a colonic resection after an episode of volvulus [8]. However, the timing of this intervention remains controversial. Moreover, those patients are usually frail and with multiple co-morbidities, making them less preferable surgical candidates. For this reason, nowadays most patients with sigmoid volvulus are not offered a surgical resection and, despite multiple admissions, are always treated conservatively, with increased risk and discomfort for the patients and high cost for the society.

Aim of this pilot study was to identify the prognostic factors to be considered in the decision-making process in patients with sigmoid volvulus and to see if a more aggressive approach early in the clinical course, as suggested by clinical evidence and guidelines, may represent a benefit for those difficult patients. This paper would represent the basis for a future prospective study.

## Materials and methods

This retrospective cohort study was performed at the Surgical Emergency Unit of the Oxford University Hospitals NHS Foundation Trust. Electronic records of patients admitted for “volvulus” in a 2-year period were reviewed. The initial screening returned 130 patients, but data of 52 patients with discharge diagnosis of caecal volvulus (24), small bowel volvulus (16), gastric volvulus (3), transverse colon volvulus (1), diverticular stricture (1) and pseudo-obstruction/ileus (7) where excluded from the analysis. The final analysis was therefore conducted on 78 patients treated for sigmoid volvulus during 332 admissions. They were 55 men and 23 women, aged 73.6 ± 14.5 (range 26–97) at index admission.

For each patient the following parameters were collected at index admission: demographic data (age, gender, social circumstances), clinical data (past medical history, comorbidities, frailty, ASA score, performance status), volvulus treatment data (eventual surgery, number of previous admissions, surgical mortality, months between first admission and surgery), radiological findings (coffee bean sign at abdominal film, CT confirmation of sigmoid volvulus, 360-degree twist, signs of ischaemia at CT), laboratory findings (white cells count, neutrophils count, c-reactive protein).

Social circumstances were coded with a “Social Score”: fully independent, supported by family, package of care, full-time care. The Clinical Frailty Scale was used to grade each patient’s frailty [9]. Performance status was graded according to the World Health Organization scale: fully active, restricted in physically strenuous activity but ambulatory and able to carry out work of a light or sedentary nature, ambulatory and capable of all selfcare but unable to carry out any work activities, capable of only limited selfcare, completely disabled and totally confined to bed or chair, dead. Social Score, Clinical Frailty Scale and WHO Performance Status are part of the initial evaluation of all our emergency patients and are documented at admission.

The outcome of each patient was initially assessed on the basis of his or her electronic records including records of consultations or investigations under other specialities. In the few cases where no recent entry was available on the electronic system, a round of phone calls was done to assess the status of the patient. For patients without a recent medical appointment and who could not be contacted by the telephone, the last entry was considered as censor date and status.

Primary endpoint of the study was actuarial survival. Secondary endpoints were the estimate of the risk of needing surgery in case of recurrent sigmoid volvulus and the identification of the factors favouring the surgical approach with the aim of providing a prognostic and decisional framework to guide the choice of the treatment on individual bases.

Data have been collected into an electronic database (MS Excel for Mac v.16.44) and analysed with two statistical packages (StatPlusMac v.7.3.32 and GNU-PSPP v.1.2.0). Distribution of each variable was initially tested for skewness and kurtosis. Comparisons of continuous variables were performed with the Student’s T-test (normally distributed variables) or with the Mann–Whitney U-test (non-parametric distribution). Comparisons of categorical variables were performed with the Pearson’s Chi-square test. Multivariate logistic regression analysis was conducted on the whole range of variables and then on those variables that had resulted most significant at univariate analysis with a stepwise approach. Survival analysis was performed with the Kaplan–Meier method and survival comparison was conducted with the log-rank test. Survival regression was conducted with the Cox Proportional-Hazards regression method. p < 0.05 was considered to be significant. Missing or incomplete data have been excluded listwise from the analysis.

Informed consent was obtained by all subjects and/or their legal guardians. No experimental protocol was applied on any of the patients. All methods were carried out in accordance with relevant guidelines and regulations.

This report has been prepared on the basis of the STROBE guidelines [10].

## Results

Fifty-eight patients (74.4%) had more than one admission for sigmoid volvulus. Average number of admissions per patient was 4.3 ± 3.9 (range 1–18, median 3). Seventy-six out of 78 patients had CT scan confirmation of volvulus: 44 had findings of volvulus without ischaemia, 32 had signs of volvulus with ischaemia and 2 had signs of ischaemia without clear signs of volvulus at CT (but confirmed at abdominal X-ray).”

Thirty-one patients underwent surgery (39.7%): Hartmann’s operation in 17 cases (12 open and 5 laparoscopic), sigmoid resection with primary anastomosis in 13 cases (7 open and 6 laparoscopic), sigmoid resection with anastomosis plus right hemicolectomy (for caecal perforation) and ileostomy in 1 case.

Univariate analysis of the factors predisposing to surgery is reported in Table 1.

**Table 1:** Univariate analysis

Factor	Overall*	Surgery**	No surgery**	*p*
Total	78	31 (39.7%)	47 (60.3%)	
Gender				
M	55 (70.5%)	18 (32.7%)	37 (67.3%)	**0.050**
F	23 (29.5%)	13 (56.5%)	10 (43.5%)	
Social score				
0	32 (42.7%)	18 (56.3%)	14 (43.7%)	0.149
1	8 (10.7%)	3 (37.5%)	5 (62.5%)	
2	14 (18.7%)	4 (28.6%)	10 (71.4%)	
3	21 (28.0%)	6 (28.6%)	15 (71.4%)	
ASA score				
1	1 (1.3%)	1 (100%)	0	0.243
2	35 (45.5%)	17 (48.6%)	18 (51.4%)	
3	34 (44.2%)	10 (29.4%)	24 (70.6%)	
4	7 (9.1%)	3 (42.9%)	4 (57.1%)	
Neurological disease				
Yes	46 (59.7%)	17 (37.0%)	29 (63.0%)	0.472
No	31 (40.3%)	14 (45.2%)	17 (54.8%)	
Frailty score				
1	7 (9.3%)	3 (42.9%)	4 (57.1%)	0.748
2	10 (13.3%)	5 (50.0%)	5 (50.0%)	
3	13 (17.33%)	7 (53.9%)	6 (46.1%)	
4	8 (10.7%)	4 (50.0%)	4 (50.0%)	
5	3 (4.0%)	1 (33.3%)	2 (66.7%)	
6	13 (17.3%)	6 (46.2%)	7 (53.9%)	
7	18 (24.0%)	5 (27.8%)	13 (72.2%)	
8	2 (2.7%)	0	2 (100%)	
9	1 (1.3%)	0	1 (100%)	
Performance status				
0	9 (11.8%)	4 (44.4%)	5 (55.6%)	0.299
1	14 (18.4%)	6 (42.9%)	8 (57.1%)	
2	13 (17.1%)	8 (61.5%)	5 (38.5%)	
3	27 (35.5%)	7 (25.9%)	20 (74.1%)	
4	13 (17.1%)	5 (38.5%)	8 (61.5%)	
Coffee bean sign at AXR				
Yes	3 (4.2%)	2 (66.7%)	1 (33.3%)	0.341
No	69 (95.8%)	27 (39.1%)	42 (60.9%)	
CT confirmed volvulus				
Yes	44 (57.9%)	15 (34.1%)	29 (65.9%)	0.392
No	32 (42.1%)	14 (43.8%)	18 (56.2%)	
360-degree twist				
Yes	19 (24.7%)	10 (52.6%)	9 (47.4%)	0.159
No	58 (75.3%)	20 (34.5%)	38 (65.5%)	
Ischaemia on CT				
Yes	35 (48.0%)	15 (42.9%)	20 (57.1%)	0.319
No	38 (52.0%)	12 (31.6%)	26 (68.4%)	
Abnormal WCC				
Yes	33 (42.3%)	11 (33.3%)	22 (66.7%)	0.322
No	45 (57.7%)	20 (44.4%)	25 (55.6%)	
Abnormal neutrophils				
Yes	32 (41.0%)	10 (31.3%)	22 (68.7%)	0.201
No	46 (59.0%)	21 (45.7%)	25 (54.3%)	
Abnormal CRP				
Yes	46 (59.0%)	13 (28.3%)	33 (71.7%)	**0.013**
No	32 (41.0%)	18 (56.3%)	14 (43.7%)	
Admissions > 3				
Yes	32 (41.0%)	19 (61.3%)	13 (27.7%)	**0.003**
No	46 (59.0%)	12 (38.7%)	34 (72.3%)	

*ASA* American Society of Anaesthesiologists, *AXR* abdominal X-ray, *CT* computed tomography, *WCC* white cells count, *CRP* C-reactive protein

*Percentage within column; **percentage within row

**Fig. 1:**
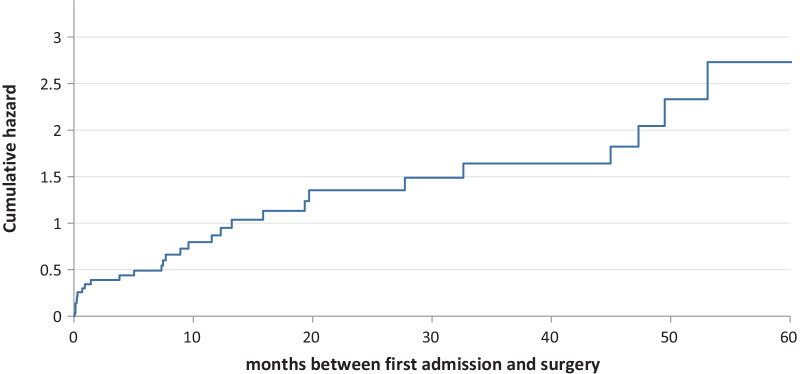
Proportional hazard to undergo surgery

Essentially, female patients and those with normal CRP are more prone to undergo surgery. Furthermore, patients are more likely to undergo a surgical operation within their first 3 admissions for volvulus. In fact, the curve of the proportional hazard to undergo surgery is particularly steep in the first phase (Fig. 1).

Multivariate analysis by binary logistic regression confirmed gender, CRP and admissions greater than 3 as independent prognostic factors (Table 2).

**Table 2:** Multivariate analysis

Factor	Beta coefficient	Odds ratio	*p*
Gender (male)	− 1.20	0.30	0.036
Admissions > 3	1.47	4.37	0.005
Abnormal CRP	− 1.12	0.33	0.033
Intercept	0.38		

Dependent variable: surgery. Overall model fit: Chi-square = 18.1 − Degrees of freedom = 3 − p < 0.001

*CRP* C-reactive protein

**Fig. 2:**
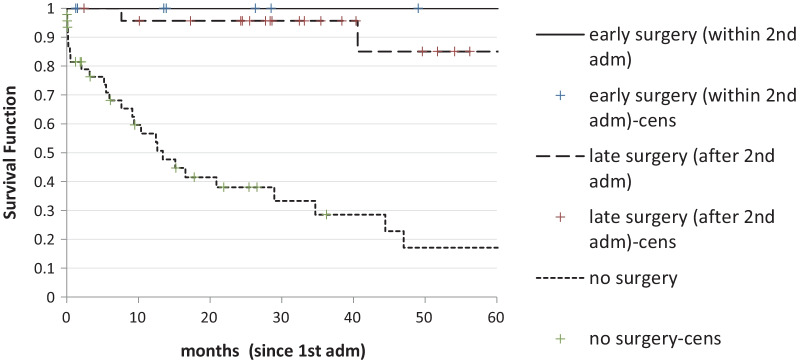
Survival according to treatment (early surgery vs late surgery vs no surgery) (p < 0.001)

**Fig. 3:**
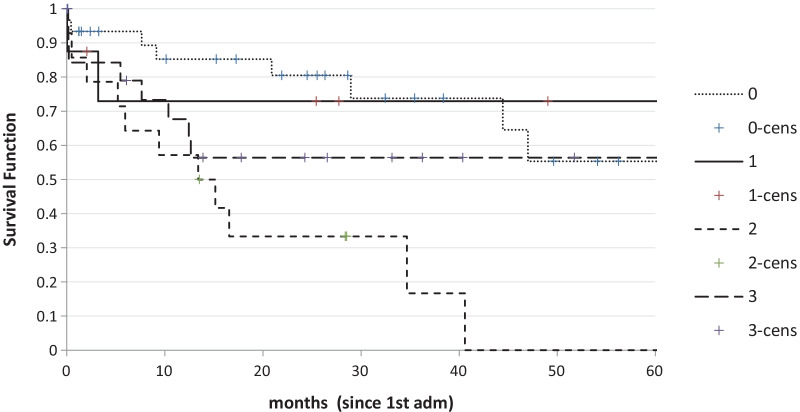
Survival according to Social Score (p < 0.01) (for definition of Social Score see text)

Median follow-up was 15.2 months (range 1–133). Overall mean survival was 54.9 ± 8.8 months since the first admission and was significantly better in patients who did have an operation than in those who did not (68.6 ± 7.2 vs 31.4 ± 7.8, p < 0.001), but did not change significantly according to timing of surgery, either within the first 2 admissions or afterwards (Fig. 2). Survival was also better in patients with low Social Score (Fig. 3).

**Fig. 4:**
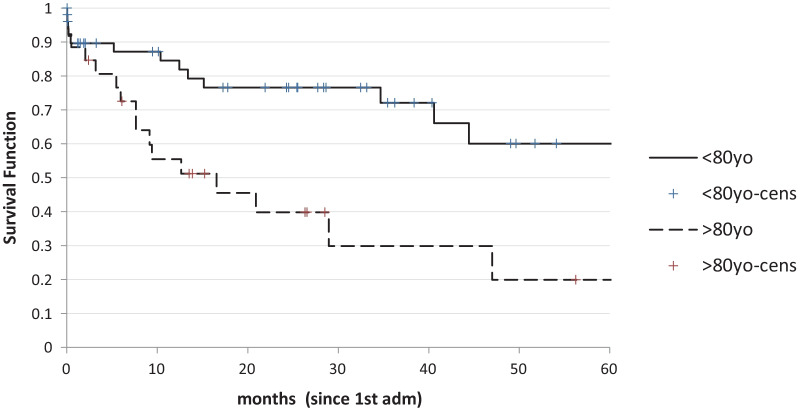
Survival according to age (> or < 80) (p < 0.003)

**Fig. 5:**
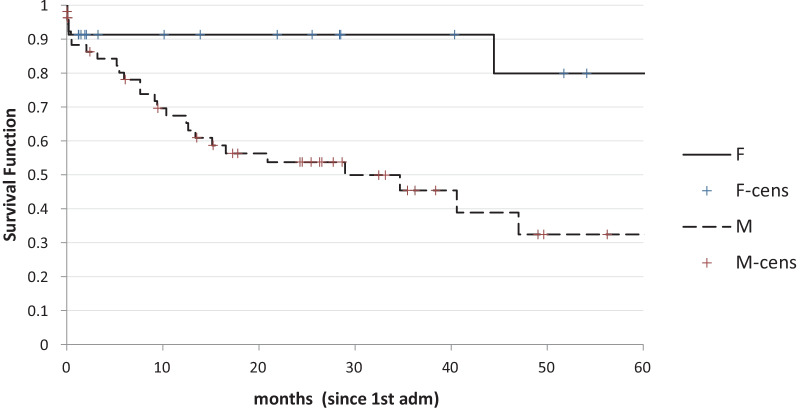
Survival according to gender (p < 0.005)

However, the actual difference is mostly in the first 12 months of the survival curve as in the long time the curves tend to converge. Survival was longer in younger patients (Fig. 4) and was worse in man with respect to women (Fig. 5).

Cox regression analysis confirmed only gender (male) and surgery as independent prognostic factors (Table 3).

**Table 3:** Multivariate analysis of survival (Cox Proportional-Hazards Regression)

Factor	Beta coefficient	*p*
Gender (male)	1.228	0.026
Surgery	− 2.012	0.0002

Overall model fit: Chi-square = 30.756 − p < 0.0001

## Discussion

Sigmoid volvulus is a frequent reason for emergency surgical admission. Despite Literature evidence and guidelines, the usual emergency treatment is bowel decompression and derotation, but at the price of a high rate of recurrence that in our series is about 75%, which is consistent with the available Literature [4–7, 11, 12]. Mortality has been shown to be up to 20% during recurrence and so endoscopic devolution must be considered only a temporary measure [7, 13] leading to a definitive treatment where possible.

Unfortunately, guidance on indications and timing of surgery are not very clear, and the decision remains with the emergency surgeon and the patient whose ability to understand, retain and use the information given may be impaired due to their chronic and acute illness.

Patients with normal CRP are more likely to undergo surgery. This finding can be related to a strict selection of patients, trying to avoid surgery in those patients with sepsis and therefore with high risk of mortality, as demonstrated by Heo et al. [14]. In fact, while for other conditions sepsis would prompt an emergency operation for source control, in already frail and looked-after patients, sepsis can be a terminal event and surgery can be considered futile.

Patients are more likely to undergo surgery within their first three admissions. This may relate to the patient’s fitness for surgery and/or to their complicated presentation. The ASCRS guidelines advised that after a single episode of volvulus elective surgery should be planned to prevent further recurrence [8], but not all patients are considered fit for surgery at their first admission. Despite the presence of objective criteria, fitness for surgery is often a matter of subjective evaluation that can be biased by personal ideas and impressions. As a matter of fact, in our series the choice of surgery was not influenced by the ASA score (Table 1), thus demonstrating that at the moment the surgical choice is still based on not-better-specified “clinical criteria”. On the contrary, we feel that surgery must be considered the first choice in patients whose fitness has been evaluated by strict evidence-based criteria, also considering the overall low surgical risk and the low risk of recurrence after surgical resection [7].

Quénéhervé et al. found that patients in their ‘no surgery group’ were older and frailer and agree that surgeons are more reluctant to carry out colonic surgery on this cohort of patients, therefore, quite expectedly, general conditions of the patients—and their frailty—may represent a factor to be considered when deciding the treatment strategy [3].

The timing of planned surgery remains controversial. Some suggest that definitive surgery should be carried out within 2–5 days of the initial volvulus [15, 16]. Furthermore Johansson et al. found that recurrence was more frequent after the second episode, leading us to believe that elective surgery should only be advised following the second recurrence and not the first [5]. Our series demonstrates that there is no significant difference of long-term survival according to timing of surgery (Fig. 2), therefore early surgery may be suggested against a late operation mostly to improve quality of life and reduce the risk of further admissions.

Long term survival in this cohort of patients strictly depends on the treatment they receive. Overall mean survival was about 5 years, but long-term survival can only be possible in patients who undergo a surgical resection, while only less than 20% of non-surgical patients are still alive 5 years after their presentation. Ifversen et al. found that patients who were treated surgically after the first occurrence had a far better survival. [4]. Interestingly, in our cohort, survival was not affected by timing of surgery and patients operated after the first two admissions had similar survival than those operated earlier. However, the actuarial curves are not completely overlapping (Fig. 2), allowing us to hypothesize that with a larger sample and a longer follow-up it could be possible to highlight an advantage for the patients who had an earlier operation.

Our study shows that survival was better in patients with low social score. It is worth specifying that our “social score” is only indirectly related to medical conditions and general frailty, being on the contrary a classification of the social circumstances of the patient. While it is obvious that - generally speaking - more frail patients may likely need a more complex social support, we wonder if this is enough to justify a shorter survival in patients with high social score, independently of clinical frailty. In fact, in our analysis, frailty has not been found to be an independently prognostic variable. It looks like some frail patients who live independently may have better outcomes with respect to those with the same frailty who need strong social support. This is an interesting issue that should be analysed with a different study design on a larger population.

In our study survival was also better in women. This may be, at least partially, related to the fact that women were more likely to be offered a surgical operation, therefore they might have been in better general conditions. However, this recalls once again the issue of ‘clinical perception’. It is possible that women were perceived to be in better general conditions, without using objective criteria but only the surgical “first impression”. Another factor must be anyway taken into account, the natural longer survival and the greater resilience of female patients with respect to men [17].

This paper offers a significant insight on a selected cohort and allows to draw interesting conclusions which somewhat challenge the current conservative attitude towards patients treated for sigmoid volvulus, supporting clinical evidence and guidelines. However, beyond its intrinsic value, it can be regarded as a pilot study that can prompt further research. In fact, main limitations of our paper are the small sample size and its retrospective nature, along with the relatively short follow-up. Although both our cohorts of surgical and non-surgical patients matched for every basic clinical aspect, the surgical decision was not always based on strict clinical criteria but mostly on the choice of the surgeon in charge. A proper randomised controlled trial would be able to clarify some still unsolved issues, such as indications, contraindications and timing of surgery.

In conclusion, a more aggressive approach to patients with sigmoid volvulus seems to be justified on the ground of the demonstration of a better and longer survival in those who undergo a surgical operation. We suggest that every patient with recurrent sigmoid volvulus is thoroughly assessed for fitness and offered surgery as soon as possible.

## Data Availability

The datasets generated during and analysed during the current study are not publicly available due to local regulations, but are available from the corresponding author on reasonable request.

## Abbreviations

ASA: American Society of Anaesthesiologists
ASCRS: American Society of Colon and Rectal Surgeons
AXR: Abdominal X-ray
CRP: C-reactive protein
CT: Computer tomography
STROBE: Strengthening the reporting of observational studies in epidemiology
WCC: White cells count
